# A peptide identification-free, genome sequence-independent shotgun proteomics workflow for strain-level bacterial differentiation

**DOI:** 10.1038/srep14337

**Published:** 2015-09-23

**Authors:** Wenguang Shao, Min Zhang, Henry Lam, Stanley C. K. Lau

**Affiliations:** 1 Division of Biomedical Engineering, The Hong Kong University of Science and Technology, Clear Water Bay, Hong Kong; 2 Division of Environment, The Hong Kong University of Science and Technology, Clear Water Bay, Hong Kong; 3 Department of Chemical and Biomolecular Engineering, The Hong Kong University of Science and Technology, Clear Water Bay, Hong Kong; 4 Division of Life Science, The Hong Kong University of Science and Technology, Clear Water Bay, Hong Kong

## Abstract

Shotgun proteomics is an emerging tool for bacterial identification and differentiation. However, the identification of the mass spectra of peptides to genome-derived peptide sequences remains a key issue that limits the use of shotgun proteomics to bacteria with genome sequences available. In this proof-of-concept study, we report a novel bacterial fingerprinting method that enjoys the resolving power and accuracy of mass spectrometry without the burden of peptide identification (i.e. genome sequence-independent). This method uses a similarity-clustering algorithm to search for mass spectra that are derived from the same peptide and merge them into a unique consensus spectrum as the basis to generate proteomic fingerprints of bacterial isolates. In comparison to a traditional peptide identification-based shotgun proteomics workflow and a PCR-based DNA fingerprinting method targeting the repetitive extragenic palindromes elements in bacterial genomes, the novel method generated fingerprints that were richer in information and more discriminative in differentiating *E. coli* isolates by their animal sources. The novel method is readily deployable to any cultivable bacteria, and may be used for several fields of study such as environmental microbiology, applied microbiology, and clinical microbiology.

Sensitive and accurate differentiation of bacteria is important not only to microbiology but also many disciplines of basic and applied sciences. DNA fingerprinting has been one of the most commonly used strategies, as it offers a good balance between detection performance and operational convenience^1^. In recent years, proteomic fingerprinting using mass spectrometry (MS) has emerged as a robust alternative^2^. By taking a snapshot of the system-wide expression profile under specific growth conditions, proteomic fingerprinting may reveal subtle differences between closely related bacterial strains that cannot be resolved by using DNA fingerprinting^2^.

Proteomic fingerprints can be generated using MS analysis of intact proteins (top-down) or digested peptides (bottom-up). Matrix-assisted laser desorption ionization (MALDI) time-of-flight (TOF) MS is a top-down technique widely used for rapid identification of clinically relevant bacteria at the genus and species levels^3^,^4^. Bacteria are identified through the matching of their MS profiles to those of previously characterized strains without involving protein identification. MALDI-TOF-MS is relatively high in throughput and easy to perform, requiring only the mixing and air-drying of intact cells in a matrix prior to MS analysis. However, its MS profile quality, mass accuracy, data richness, and reproducibility have yet to be improved so as to deliver the resolution and discriminative power required for consistent bacterial differentiation at the strain level^4^.

Contrarily, shotgun proteomics is a bottom-up technique that involves tryptic digestion of the total proteins extracted from cell lysate, followed by liquid chromatography (LC) coupled with tandem mass (MS/MS) spectrometry analysis of the peptide mixture^5^,^6^. Bacterial differentiation and identification typically involve the assignment of MS/MS spectra to peptide sequences that are in silico digested from protein sequences translated from publicly available bacterial genome sequences^7^,^8^. This technique has been used successfully in differentiating closely-related strains of *Helicobacter pylori* as well as pathogenic strains of *E. coli* and *Yersinia pestis* from non-pathogenic ones^2^,^9^.

LC-MS/MS offers high detection sensitivity and high dynamic range, resolving thousands of peptide species that vary widely in abundance within a sample^10^. However, the unambiguous assignment of MS/MS spectra to peptides ultimately depends on the availability of genome sequences of the bacteria concerned^11^,^12^. The term proteogenomics inscribes the strong links between genome sequence data and protein/peptide identification^13^. Although the number of genome sequences available in the public domains has been increasing at unprecedented rates, there remain many bacterial species yet to be sequenced. For species that have been sequenced, a species is often represented by the genome sequence(s) of only one or a few representative strains. This may undermine the vast gene pool that is present in the pangenome of a bacterial species^14^ and may limit the resolving power of shotgun proteomics for strain-level differentiation and identification.

Recently, a novel peptide identification-free, and thus genome sequence-independent, shotgun proteomic workflow has been developed^15^. This workflow uses a similarity-clustering algorithm to group MS/MS spectra that are likely derived from the same peptide ion and subsequently merge them into a unique consensus spectrum as the basis to generate proteomic fingerprints. The workflow has been used successfully in fingerprinting the remnant blood meals in the blacklegged tick (*Ixodes scapularis*), and tracing the blood meal to the vertebrate host that a tick had last fed on.

In this study, we tested this peptide identification-free proteomics workflow as a novel fingerprinting method for bacterial differentiation and identification (referred to as UNID-proteomic fingerprinting hereafter). The test model was comprised of *E. coli* isolates pertaining to four species of animal hosts of contrasting diet and habitat. *E. coli* is prevalent in the enteric environments of mammalian hosts. Nonetheless, each animal species features a unique combination of diet, gut anatomy, physiology, and behavior that shape the conditions of its enteric environment, which in turn, enriches for a different diversity of *E. coli* populations^16^. The tracing of *E. coli* isolates to animal sources has important implications to epidemiology and the control of food and water hygiene^17^. We evaluated the accuracy and sensitivity of UNID-proteomic fingerprinting in differentiating and identifying the animal sources of the *E. coli* isolates in reference to those of a conventional proteomic workflow that involved peptide identification^18^ (ID-proteomic fingerprinting hereafter) and a typical PCR-based DNA fingerprinting method that targeted the repetitive extragenic palindromes (REP) elements in bacterial genomes^19^,^20^. The procedures, working principal, performance, advantages, limitations, and potential application of UNID-proteomic fingerprinting are discussed below.

## Methods

### *E. coli* isolates

Eighty *E. coli* isolates were obtained from four sources (raw sewage, and the freshly voided feces of feral cows, pet dogs and farm pigs) (Table 1). Sewage was collected from the soil pipe of a pet-free university dormitory and hence assumed to be from humans exclusively. The isolation was performed using CHROMagar™ ECC, which is designed to suppress the growth of Gram-positive bacteria, and to differentiate *E. coli* from other Gram-negative bacteria by the formation of blue colonies as a result of β-d-galactosidase and β-d-glucuronidase activities^21^. The blue colonies isolated as putative *E. coli* were verified for identity using two different PCR assays. One was a multiplex PCR targeting the gene that encodes a respiratory oxidase in *E. coli* (the cytochrome bd complex), and the genes that encode the enzymes whose activities would lead to the blue coloration of *E. coli* colonies on CHROMagar^TM^ ECC (lactose permease, β-d-galactosidase and β-d-glucuronidase)^22^. The other PCR method targeted a ca. 544 bp fragment of the 16S rRNA gene specific to *E. coli*^23^. Isolates that were detected positive for all five genes were stocked at −80 °C until use. The primers of the two PCR assays are in Supplementary Table S1.

**Figure 1:**
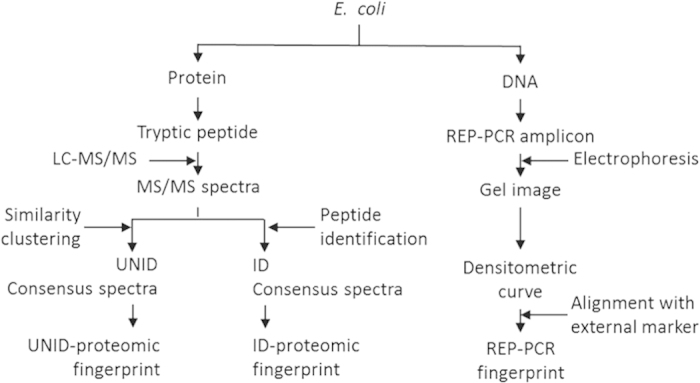
Flowchart for the major steps in sample preparation, data acquisition, and data analysis involved in UNID-proteomic, ID-proteomic, and REP-PCR fingerprinting.

*E. coli* isolates were cultured to early stationary phase and washed to obtain cell pellets for DNA and protein extraction (Fig. 1) as described in the Supplementary Methods. However, the stocks of seven isolates (six of pig and one of dog) became non-cultivable during the course of this study, as they showed no growth in the culture medium in repeated attempts. There remained 73 isolates to complete the study (Table 1).

### LC-MS/MS analysis

Each cell pellet was lysed and it had protein digested with trypsin as detailed in the Supplementary Methods. Tryptic digest eluted in 0.1% formic acid (FA) in water was injected to an LTQ Velos dual-pressure ion trap mass spectrometer (Thermo Fisher Scientific) that was interfaced to a nano-electrospray ion source (spray voltage 1.6 kV, capillary temperature 250 °C), a Thermo Accela 600 pump, and an Accela autosampler LC. A BioBasic-18 column was used (dimension: 150.0 × 0.1 mm, particle size: 5 μm; Thermo Scientific). Solvents A and B in the mobile phase (flow rate: 150 μl min^−1^) were 0.1% FA in water and 0.1% FA in acetonitrile, respectively. The chromatographic conditions and data acquisition method are described in the Supplementary Methods. LC-MS grade water and acetonitrile were from J. T. Baker. All other supplies and reagents were from Sigma-Aldrich, unless specified.

### Proteomic fingerprinting without peptide identification

One run of LC-MS/MS analysis was performed for each *E. coli* isolate (Fig. 1). The MS/MS spectra of all isolates were compiled into a spectral dataset and converted into the mzXML format using msConvert^24^.

Then, the MS/MS spectra were subjected to a quality-checking algorithm using SpectraST^25^. MS/MS spectra would pass the quality check when having: (i) ≥35 fragments, (ii) a precursor m/z >350 Th, (iii) the m/z of the heaviest and the lightest fragment differed by >350 Th, and (iv) >5% of the total intensity represented by fragments that were larger than the precursor m/z. Criterion four served to differentiate peptide ions (typically multi-charged upon electrospray ionization) from non-peptide ions (typically single-charged). MS/MS spectra passing the quality check were analyzed using the similarity-clustering algorithm of SpectraST^15^,^25^. MS/MS spectra that had (i) precursor *m/z* differed by <2.5 Th, and (ii) pairwise dot product similarity >0.7 were regarded as replicate spectra derived from multiple observations of the same peptide ion species, and were hence merged into a unique consensus spectrum (UNID-consensus spectrum hereafter) after peak alignment and noise-filtering (Fig. 1).

These procedures resulted in a spectral library that contained entries of individual UNID-consensus spectra along with the number and origin (i.e. which *E. coli* isolate) of the replicate spectra constituting each of them. Then, for each isolate, the numbers of replicate spectra that constituted its individual UNID-consensus spectra were normalized using the total number of MS/MS spectra acquired for the isolate. The collection of UNID-consensus spectra (i.e. presumably each represented a different peptide ion species) and the normalized abundance of the constituting replicate spectra (i.e. relative spectral counts of individual presumptive peptide ion species) associated with each isolate were used as the proteomic fingerprint (UNID-proteomic fingerprint hereafter) of the isolate (Fig. 1).

### Proteomic fingerprinting based on peptide identification

The MS/MS spectral dataset was also subjected to peptide identification (Fig. 1). A protein database was constructed by combining all reviewed *E. coli* proteins in the UniProt-SwissProt database, and the common contaminant proteins listed in the common Repository of Adventitious Proteins. Then, the protein database was appended to an equal-size decoy database generated by random shuffling of amino acids between tryptic sites^26^. Two traditional sequence database searching engines, namely OMSSA (ver 2.1.8)^27^ and X!Tandem with K-score plugin (ver 2011.12.01.1)^28^, were used with parameters as follows: (i) trypsin as the digestion enzyme, (ii) allowing the missing of one internal tryptic cleavage, (iii) carbamidomethylation on cysteines as a fixed modification, (iv) oxidation of methionine as a variable modification, (v) mass tolerances of +/−3 Da for precursors, and (vi) +/−1 Da for product ions. Refinement search was enabled in X!Tandem in an error tolerant mode, allowing the identification of single nucleotide polymorphism.

The search results were analyzed and combined using PeptideProphet and iProphet^29^ in the Trans Proteomic Pipeline (v4.5.3)^30^. Upon filtering with a global false discovery rate of 1%, as estimated by a target-decoy strategy, MS/MS spectra that were identified to the same peptide were merged into a unique consensus spectrum (ID-consensus spectrum hereafter) after peak alignment and noise-filtering. For each *E. coli* isolate, the collection of ID-consensus spectra and the relative abundance of the constituting MS/MS spectra were used as its ID-proteomic fingerprint (Fig. 1).

### Interspersed repetitive extragenic palindromes (REP)-PCR fingerprinting

DNA was extracted from cell pellet as described in the Supplementary Methods. REP sequences were PCR-amplified using primers Rep1R-I (5′-ICGICGICATCIGGC-3′) and Rep2-I (5′-ICGICTTATCIGGCCTAC-3′) and resolved using gel electrophoresis as described previously^31^. On each 15-lane gel, the first and the last lanes were not used so as to minimize intra-gel distortion of fingerprints whereas the 2^nd^, 8^th^, and 14^th^ lanes were loaded with a 1-Kb DNA ladder. Images of REP-PCR fingerprints captured from the gels after ethidium bromide staining were transformed into densitometric curves and aligned to that of the 1-Kb DNA ladder using Bionumerics® version 6.5 (Applied Maths) (Fig. 1).

### Data analysis

All analyses were conducted in R^32^ with the “ape”^33^ or “mixOmics” package^34^. *E. coli* isolates were compared pairwise for similarity in each fingerprinting type using Pearson correlation coefficient. The similarity matrices were visualized using multidimensional scaling (MDS). Differences among the four source groups (sewage, cow, dog and pig) were tested using the analysis of similarity (ANOSIM). Jackknife analysis was used as an internal measure of the accuracy of a fingerprint library in classifying a query isolate to the correct source^35^. For each source group, the percentage of its isolates being correctly assigned to the group was determined as the rate of correct classification (RCC). An average RCC (ARCC) was determined for all source groups in a library.

Since UNID-proteomic fingerprinting did not involve peptide identification, selected UNID-consensus spectra were computationally evaluated for their likelihood of being derived from peptides. Briefly, partial least squares discriminant analysis (PLS-DA) was conducted to rank the UNID-consensus spectra for their relative importance (referred to as “loading” in PLS-DA terminology) in differentiating *E. coli* isolates by source group^36^. For each source group, UNID-consensus spectra of the top ten highest loading were subjected to *de novo* sequencing to deduce a peptide’s amino acid sequence from its MS/MS spectra without searching a protein database^37^,^38^. *De novo* sequencing was conducted with PEAKs studio (version 7.0; build 20131119)^39^ with default parameters, except that: i) parent mass error tolerance was 1.5 Da, ii) fragment mass error tolerance was 0.5 Da, iii) trypsin as digestion enzyme, iv) carbamidomethylation (+57.02 Da, C) as fixed modification, v) oxidation (+15.99 Da, M) as variable modification, and vi) maximum variable post-translation modification allowed per peptide was three. Sequence tags were identified with at least one b or y ion matched within the fragment mass error tolerance.

### Accession number

The mass spectrometry proteomics data have been deposited to the ProteomeXchange Consortium^40^ via the PRIDE partner repository with the dataset identifier PXD001646.

## Results and Discussion

### UNID- and ID-consensus spectral libraries

**Figure 2:**
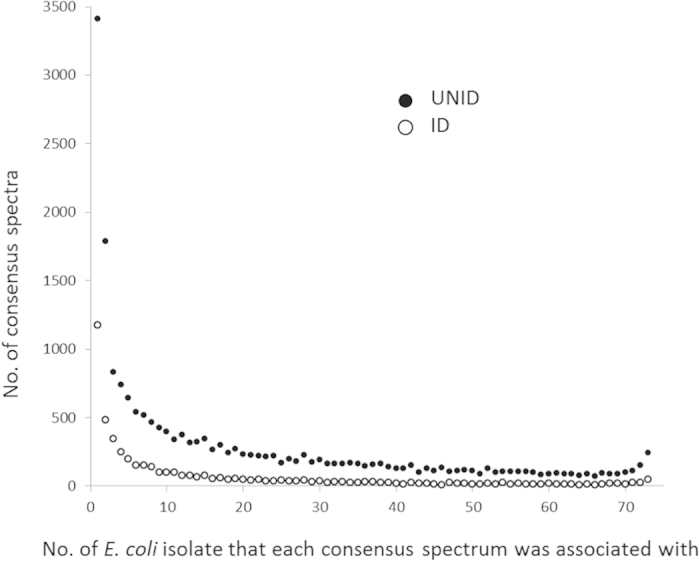
The ubiquity of the UNID- and ID-consensus spectra obtained. Data shown are the number of consensus spectra that belonged to each level of ubiquity, from being associated with only one isolate tested to being associated with all 73 isolates tested.

A total of 1,188,790 MS/MS spectra were obtained from the tryptic digest of the 73 *E. coli* isolates. Using the similarity-clustering algorithm, 61% (734,723) of the MS/MS spectra were merged into 19,950 UNID-consensus spectra; the remaining ones were discarded for being regarded as low-quality or non-peptide derived. On the contrary, the conventional workflow of peptide identification could made use of only 11.9% (142,376) of the MS/MS spectra and merged them into 5,025 ID-consensus spectra. The consensus spectra (both UNID and ID) varied in ubiquity, from being associated with only one isolate to being associated with all isolates tested (Fig. 2). There were 3,414 UNID-consensus spectra and 1176 ID-consensus spectra being isolate-specific. The numbers decreased as the ubiquity of the consensus spectra increased, except for those that were almost universally present in all isolates tested (i.e. ≥70 isolates) (Fig. 2), resembling the U-shaped distribution of gene frequency typically observed for the pangenome of a bacterial species^41^,^42^. Such distribution was more obvious for the UNID-consensus spectra than the ID counterparts (Fig. 2).

*E. coli* exhibits a very high level of genome plasticity^43^. The sizes of the >2,400 genome sequences of *E. coli* currently available in the Genbank differ by as much as 2 Mb, varying between 4.0 and 5.9 Mb. Among the ~16,000 homolog gene clusters that have been identified for the *E. coli* pangenome, only 10% belong to the core genome; the remaining ones distribute variably among strains^44^. Despite the intense sequencing efforts, the pangenome of *E. coli* is far from saturation^41^,^45^, implying that the MS/MS spectra of many peptides that are rare or potentially strain-specific are not readily identifiable.

Indeed, our results indicate that the genome sequence-independent similarity-clustering algorithm utilized the MS/MS spectra to a fuller extent than the peptide identification workflow did, and yielded a total number of UNID-consensus spectra that was nearly four times of that of the ID counterparts. In particular, there were large differences between the numbers of UNID- and ID-consensus spectra that were sparsely distributed among isolates (Fig. 2). We suggest that, by cutting free the requirement for peptide identification, the UNID consensus spectra constituted a presumably more informative representation of the proteomes of the isolates tested, particularly for peptides that were potentially isolate-specific.

### Differentiation of *E. coli* isolates by animal sources

On average, each *E. coli* isolate tested in this study had 5,238 UNID- and 1,013 ID-consensus spectra in its two proteomic fingerprints, respectively. Contrarily, the REP-PCR fingerprint of each isolate was a densitometric curve of a gel lane that had only 15–25 visually discernible bands. This stark contrast in information richness stemmed from not only the inherent differences in biological nature of the analytes (further discussed below) but also the ability of LC-MS/MS to survey its analytes in greater depth. In spite of the substantial differences in information richness, the analysis of the two proteomic (UNID and ID) fingerprints and the REP-PCR fingerprints consistently indicated that *E. coli* isolates of the same source group were generally closer related to each other than were isolates between groups (p < 0.001, ANOSIM; Table 2). UNID-proteomic fingerprinting resulted in the largest degrees of between-group separation (global R-value: 0.814), followed by ID-proteomic (global R-value: 0.735) and REP-PCR fingerprinting (global R-value: 0.640) (Table 2).

**Figure 3:**
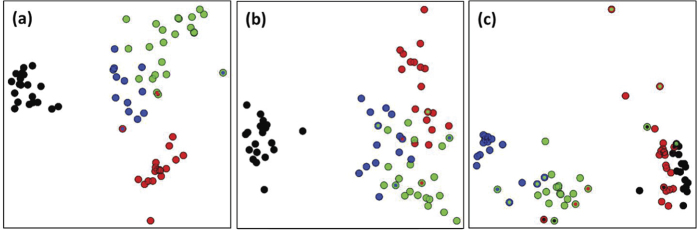
Multi-dimensional scaling of 73 *E. coli* isolates on the basis of UNID-proteomic (a), ID-proteomic (b) and REP-PCR fingerprints (c). Each dot represents a different isolate. Dots in green, black, red and blue represent isolates of sewage, cow, dog and pig, respectively. Dots with an outline of a different color indicate that the isolates were misclassified in Jackknife analysis. The color of the outline indicates the source group that the isolate was misclassified to.

MDS ordination of the UNID-proteomic fingerprints placed the 73 isolates into three clusters that largely conformed to the dietary requirement of the animal hosts (Fig. 3a). One cluster consisted all isolates of cow (ruminant). Another one contained all but two isolates of dog (carnivore). The isolates of pig and sewage (omnivorous) formed a third cluster that also contained two isolates of dog. Many other studies have also reported that some clones of *E. coli* were less host-species and that they could be found in association with multiple species of animal hosts^46^,^47^. The ID-proteomic fingerprints resulted in a similar MDS ordination but a less clear segregation of the isolates by source groups (Fig. 3b). Contrarily, the MDS ordination of REP-PCR fingerprints put most isolates of cow and dog into a single cluster (Fig. 3c); this contradicts the gut anatomy and diet of dog being closer related to those of human and pig than to cow.

REP-elements are 21–65 bases long non-coding sequences, occupying >0.5% extragenic space throughout a bacterial genome^48^,^49^. Because REP-elements are typically located between convergent genes, their distribution patterns within genomes, as shown in the PCR-fingerprints, can reflect evolutionary relationship between bacteria^48^,^49^. Contrarily, shotgun proteomics takes a snapshot of the system-wide expression of bacterial cells under a particular growth condition. LC-MS/MS is able to resolve peptides that differ by as little as one amino acid, allowing the differentiation of protein variants that are produced by different strains of a bacterial species^12^. Such protein variants, as a result of homologous recombination or point mutation, can have important implications to intraspecific diversification and adaptation to different habitats^50^.

As far as the *E. coli* isolates tested in this study are concerned, source differentiation by proteomic fingerprinting appeared to be ecologically more relevant than by REP-PCR fingerprinting (Fig. 3). The genetic structure of *E. coli* populations in animal hosts is shaped by a multitude of host and environmental factors^47^. Although *E. coli* is regarded as a clonal species, both homologous recombination and mutation play important roles in shaping its genetic structure^43^,^51^,^52^. In addition, the extensive acquisition and loss of genes (i.e. high genome plasticity) result in highly diverse adaptive paths within the species^43^. We hypothesize that the observed performance of proteomic fingerprinting in source differentiation of the *E. coli* isolates is partly due to the differentiation of isolate-specific proteins and partly the differentiation of protein variants that are associated with host adaptation. The UNID-proteomic fingerprints, which constituted a presumably fuller representation of the proteome, provided a clearer distinction of *E. coli* isolates than the ID counterparts.

### Accuracy of classification to animal sources

ARCC and RCC are often used as metrics to predict the performance of a classification system in assigning a novel case to the correct group^35^,^53^. In this study, ARCC and RCC values were calculated for each proteomic fingerprint library in 72 iterations, starting with the complete set of UNID- or ID-consensus spectra followed by successive filtering of the consensus spectra according to the number of isolates that they were each associated with (Fig. 2). This served to compare consensus spectra of different levels of ubiquity for their relative importance in source classification. Contrarily, only one set of ARCC and RCC values was calculated for REP-PCR fingerprint library because the bands in the gel lane of each isolate were transformed into a single densitometric curve instead of serving as discrete units that would allow successive filtering.

**Figure 4:**
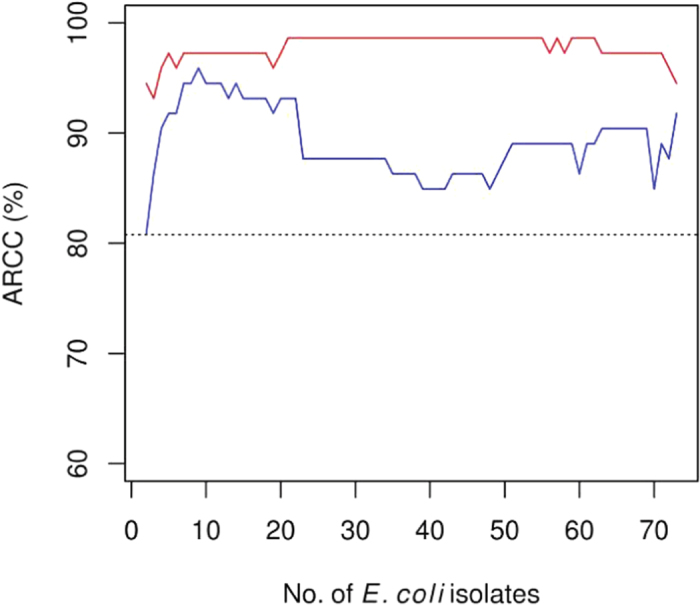
Average rate of correct classification (ARCC) of *E. coli* isolates by UNID-proteomic (red line) and ID-proteomic (blue line) fingerprint libraries. Data shown are the change in ARCC of each library as its consensus spectra were filtered according to the no. of *E. coli* isolates that they were associated with. The ARCC of REP-PCR fingerprint library (dotted line) is for reference.

ARCC of the UNID-proteomic library was 94.5% when the entire set of consensus spectra was used (Fig. 4). All isolates of cow and sewage were correctly classified (i.e. 100% RCC) whereas two dog isolates (89.5% RCC) and two pig isolates (85.7% RCC) were not (Table 3 & Supplementary Table S2). All RCC values were larger than the 25% probability of having correct classification merely by chance in a 4-way split (sewage vs. cow vs. dog vs. pig)^35^. These results were largely congruent to the MDS ordination where the misclassified isolates were positioned closer to isolates of other sources than those of their own (Fig. 3a). An exception was a pig isolate that was misclassified to dog; this isolate clustered with other pig isolates in the MDS ordination (Fig. 3a).

The filtering of UNID-consensus spectra that were associated with all 73 isolates (i.e. retaining those associated with ≤72 isolates) resulted in the raise of ARCC to 95.9% (Fig. 4). In further filtering, ARCC increased to the highest of 98.6% (retaining those associated with ≤62 isolates) and remained largely unchanged until the UNID-consensus spectra retained were each associated with ≤23 isolates. After that, ARCC declined to the lowest of 93.2% when the UNID-consensus spectra retained were each associated with ≤3 isolates. Even after such extensive removal, the ARCC remained higher than that of the REP-PCR fingerprint library (80.8%), indicating the strongly discriminating features of the UNID-consensus spectra being investigated (Fig. 4).

In line with the less conspicuous distinction of *E. coli* isolates by source groups (Fig. 3b), the ID-proteomic fingerprint library attained generally lower ARCC values than the UNID counterpart did (Fig. 4, Table 3 & Supplementary Table S2). Although ARCC increased as the relatively ubiquitous ID-consensus spectra were filtered, a rather extensive filtering (retaining those associated with ≤20 isolates) was required for a clear effect. ARCC values dropped rapidly as ID-consensus spectra that were associated with ≤10 isolates were successively filtered. Nonetheless, the lowest ARCC value attained (82.2%) remained higher than that of the REP-PCR counterpart (Fig. 4).

The two proteomic fingerprinting workflows differentiated and classified isolates on the basis of the presence/absence of individual consensus spectra and also the numbers of replicate MS/MS spectra constituting the consensus spectra. The latter reflected approximately the relative abundance of the underlying peptides in each protein sample. As exemplar results, the ten most influential UNID-consensus spectra that differentiated the isolates of dog from other source groups were each associated with 84.2–89.5% of the dog isolates and were completely absent from other groups (Table 4). Contrarily, UNID-consensus spectra that differentiated the isolates of cow, pig or sewage were not entirely specific as they could be found associated with up to 50% of the isolates of one or more than one source group. When an UNID-consensus spectrum that distinguished one source group was also present in the isolates of another group, the average number of replicate MS/MS spectra produced by each isolate of the former (1.5–6.5) was usually higher than the latter (1–3.6) (Table 4). *de novo* sequencing inferred prominent sequence tags for the top 10 most discriminating UNID-consensus spectra of each source group, suggesting that they were likely originated from peptides (Table 4). However, none of these consensus spectra could be confidently identified to any peptide with traditional sequence database searching engines, suggesting that these unique signatures are unlikely to be found in the protein sequences of *E. coli* in SwissProt.

### Key advantages and challenges of UNID-proteomic fingerprinting

We suggest that the generally higher performance of shotgun proteomics over REP-PCR fingerprinting was associated with the biological nature of the proteome as analyte, the accuracy of LC-MS/MS in mass detection, and the information richness of the proteomic fingerprints, as discussed in above. We further suggest that consensus spectra as discrete units comprising the proteomic fingerprints should allow a more reliable determination of fingerprint similarity between isolates than what the alignment of densitometric curves of DNA fingerprints could offer.

In spite of these advantages, the requirement of genome-derived peptide sequences for the identification of measured peptide data (i.e. MS/MS spectra) remains a key issue that limits the use of shotgun proteomics to bacteria that are genome-sequenced. Recently, shotgun proteomics has been used in conjunction with metagenomics for strain-level characterization of uncultivated bacterial communities^54^. Although the method can be used on genome-uncharacterized environmental samples, it still requires the metagenomic sequences of reference samples for peptide identification. In this proof-of-concept study, we demonstrated the possibility to use shotgun proteomics to differentiate and classify bacteria without involving peptide identification. Despite the rapidly increasing number of genome sequences being available, vast majority of the bacterial species (including those that they are yet to be cultivated or described) remain unsequenced. In principle, UNID-proteomic fingerprinting is readily deployable to any cultivable bacteria disregard of the availability of their genome sequences.

Although the *E. coli* genome is relatively well sequenced, the similarity-clustering workflow yielded a much larger number of consensus spectra than the peptide identification workflow did (Fig. 2), and the UNID-proteomic fingerprints allowed a clearer source differentiation of the *E. coli* isolates than the ID counterparts (Fig. 3). Indeed, the most discriminating UNID-consensus spectra of each animal source were not identifiable to any peptide sequence of *E. coli* (Table 4). This suggests the advantage of UNID-proteomic fingerprinting for strain-level differentiation of a genome-sequenced bacterial species that exhibits a high level of genome plasticity.

Since UNID-proteomic fingerprinting was able to differentiate the animal source of *E. coli* isolates in an ecological relevant manner (Fig. 3), we suggest that the method can be used in environmental microbiology studies in complementary to genome-based analyses to meet the needs at different stages of an investigation. For instance, UNID-proteomic fingerprinting may be used on bacteria that have been isolated from temporally and/or spatially heterogeneous (micro-) habitats for the differentiation of strains are potentially adapted to different (micro-) niches without prior knowledge of the genotypic or phenotypic attributes required for adaptation. UNID-proteomic fingerprinting may also be used in clinical microbiology for pathotyping, and in biotechnology for the screening and classification of closely related bacterial strains that potentially produce different classes of bioactive compounds.

MADLI-TOF is also an identification-free proteomic fingerprinting method. As a method that differentiates intact proteins by mass, MADLI-TOF has been used for the identification of protein markers to distinguish Shiga-toxin producing *E. coli* from non-toxigenic ones^55^, and to distinguish *E. coli* from *Salmonella* and *Acinetobacter* isolated from sewage^56^. Although MALD-TOF is high-throughput and relative easy to perform, it can detect no more than a few dozen proteins in a single sample and is unable to resolve proteins with small differences in mass (e.g. point mutations or post-translational modifications). These limitations reduce the sensitivity and accuracy in differentiating closely related bacterial strains. Previously, MALDI-TOF yielded a 73% accuracy in the classification of 15 *E. coli* isolates by animal sources (human, cow, dog and three avian species)^57^.

Shotgun proteomics is much more demanding than MALDI-TOF and DNA fingerprinting in the time, effort and expertise required for sample preparation, sample analysis and data processing. In this study, one hour of LC-MS/MS machine time was required for each *E. coli* isolate, and the throughput was one sample at a time for each machine. To construct UNID-proteomic fingerprints, MS/MS spectra were compiled into consensus spectra using spectral matching but not peptide identification. The reliability of this fingerprinting method ultimately depends on the effectiveness of the quality filtering algorithm to detect and eliminate MS/MS spectra derived from non-peptides, and also the sensitivity and specificity of the clustering algorithm to find the MS/MS spectra of one peptide and distinguish them from those of another. The clustering algorithm used in this study was previously demonstrated as being able to merge MS/MS spectra that originated from the same peptide (of ticks’ blood meals) with 95% accuracy. In this study, the performance of the clustering algorithm in handling the MS/MS spectra of bacterial peptides was indicated by the prominent sequence tags that were inferred for the top 10 most discriminating UNID-consensus spectra of each source group, suggesting the likelihood of those UNID-consensus spectra being peptide-derived (Table 4).

DNA fingerprints are invariants of the metabolic or physiological conditions of the bacterial cells being investigated. Contrarily, bacterial protein expression profiles are susceptible to batch-to-batch variations in culture conditions. In this study, we repeated the analysis of isolate classification by substituting the UNID- and ID-proteomic fingerprint libraries with the MS/MS spectra generated from biological and technical replicates of a subset of isolates (as detailed in the Supplementary Methods). Our results, as described in Supplementary Results and Discussion, indicate that UNID-proteomic fingerprinting had generally higher reproducibility in the number of consensus spectra generated and in the subsequent classification accuracy than what was observed for the ID counterpart.

The proteome of a bacterial culture also varies drastically with the growth phase^58^. All *E. coli* cultures used in this study were grown to the early stationary phase before protein extraction. It was shown for a number of bacteria, including *E. coli*, that cells at the end of the log phase would start reconstructing their proteome so as to switch the metabolism and physiology from the mode of rapid proliferation in a resourceful environment to the mode of preservation in the stressful conditions of nutrient depletion and waste accumulation in the stationary phase^58^,^59^,^60^. Therefore, the proteome of the cells in the stationary phase is believed be more relevant to adaptation than that in the log phase^58^,^59^. In this study, we tested the cultures in the early but not a later stage of the stationary phase so as to minimize the impacts of dead and decaying biomass that would accumulate over time. We have yet to test whether the performance and reproducibility of UNID-proteomic fingerprinting in bacterial differentiation and identification would vary with the growth phase of the cultures. Strict control and standardization of bacterial cultivation conditions, sample preparation procedures, and sample analysis methods are needed to enhance the reproducibility and portability of shotgun proteomics data.

DNA sequence-based typing such as multi-loci sequence typing (MLST) offers very high data reproducibility and portability, due to the unambiguous nature of DNA sequence data^61^. MLST typically involves the DNA sequences of the internal fragments of four to ten housekeeping genes, representing <0.5% of the size of a bacterial genome, although it is increasingly common to extend the analysis to the entire core genome of a species^62^. While MLST is effective in strain differentiation and identification by ancestry, it does not resolve closely related strains with genotypic differences that are associated with the gain or loss of accessory genes^63^. More recently, genome-wide mapping of single-nucleotide polymorphism and insertion/deletion has emerged a powerful alternative to MLST, revealing microevolution not only in the core genome but also the accessory genome^64^. However, a high quality full genome sequence is required as a reference for mapping. In contrast, UNID-proteomic fingerprinting has the advantages of allowing bacterial strains to be compared for differences in system-wide expression profiles in a genome sequence-independent manner. Nonetheless, the performances of UNID-proteomic fingerprinting and DNA sequence-based typing in bacterial strain differentiation and identification have yet to be compared.

### Concluding remarks

In this study, shotgun proteomics was tested for the first time for its utility in bacterial differentiation without involving peptide identification. The similarity-clustering algorithm used was previously shown to be able to generate proteomic fingerprints that allowed the blood meal of a tick to be traced to one of the 24 species of phylogenetically diverse vertebrate hosts tested (mammal, avian and reptile)^15^. In this study, we demonstrated that the similarity-clustering algorithm was also effective for generating proteomic fingerprints for the differentiation of closely related bacteria. Another novelty demonstrated in this study is the tuning of the performance of shotgun proteomics fingerprint libraries through the filtering of consensus spectra by ubiquity (Fig. 4).

In the long run, the similarity clustering workflow in this study may allow the identification of biomarkers for bacterial identification. As we have demonstrated, the UNID-consensus spectra that discriminated *E. coli* isolates by animal sources were traceable to putative peptide sequences through *de novo* sequencing (Table 4). The putative peptides, upon verification by western blot, can be evaluated for specificity as biomarkers and subsequently used as targets for more efficient MS-based quantitative proteomics assays such as selected reaction monitoring^65^. In principle, selected reaction monitoring can be used in the detection of biomarkers quantitatively not only in the proteome of pure isolates but also the metaproteome of environmental samples for cultivation-free, quantitative tracking of specific bacterial populations in manmade or natural ecosystems.

## Additional Information

**Accession codes:** The mass spectrometry proteomics data have been deposited to the ProteomeXchange Consortium via the PRIDE partner repository with the dataset identifier PXD001646.

**How to cite this article**: Shao, W. *et al.* A peptide identification-free, genome sequence-independent shotgun proteomics workflow for strain-level bacterial differentiation. *Sci. Rep.* **5**, 14337; doi: 10.1038/srep14337 (2015).

## Supplementary Material

Supplementary Information

## Figures and Tables

**Table 1:** Fecal sources of *E. coli* isolates used in this study.

Source	Collection site (all in Hong Kong)	# of samples	# of isolates	
			Collected	Tested
Sewage	Soil pipe, Hong Kong University of Science & Technology	2	20	20
Cow	Country park, Sai Kung	6	20	20
Dog	Pet shop, Tuen Mun	4	20	19
Pig	Farm, Yuen Long	4	20	14
			Total	73

Each sewage sample was collected on a different day, and each fecal sample was collected from a different individual. Originally, 20 isolates were collected from the samples of each source. But some isolates of the dogs’ and pigs’ samples lost their viability in the frozen stock. Therefore, fewer isolates were tested for those two sources.

**Table 2:** ANOSIM comparison of UNID-proteomic, ID-proteomic, and REP-PCR fingerprints among *E. coli* isolates of different source groups.

Source group	UNID-Proteomic			ID-Proteomic			REP-PCR		
	Sewage	Cow	Dog	Sewage	Cow	Dog	Sewage	Cow	Dog
Pig	0.572	0.850	0.758	0.451	0.744	0.693	0.539	0.929	0.982
Dog	0.764	0.997		0.666	0.981		0.647	0.375	
Cow	0.979			0.950			0.514		
Global			0.814			0.735			0.640

Results shown are the R-values of ANOSIM for global and pairwise comparison. The R-values vary between 0 and 1, where 0 represents that the groups concerned were identical and 1 represents that the groups were completely different. All R-values shown are statistically significant (p < 0.001).

**Table 3:** Rate of correct classification by UNID-proteomic, ID-proteomic, and REP-PCR fingerprint libraries.

Source group	UNID-Proteomic				ID-Proteomic				REP-PCR			
	Sewage	Cow	Dog	Pig	Sewage	Cow	Dog	Pig	Sewage	Cow	Dog	Pig
Sewage	100	—	—	—	90.0	—	5.0	5.0	70.0	5.0	10.0	15.0
Cow	—	100	—	—	—	100	—	—	10.0	75.0	15.0	—
Dog	10.5	—	89.5	—	5.3	—	89.5	5.3	10.5	—	89.5	—
Pig	7.1	—	7.1	85.7	14.3	—	—	85.7	7.1	—	—	92.9

Data shown in rows indicate for each source group the percentage of its isolates being assigned to its own group (i.e. correct classification) and to each of the three other groups (i.e. incorrect classification) (“−” =0%). Data for UNID- and ID-proteomic fingerprints are on the basis of the complete set of consensus spectra (i.e. no filtering; see Fig. 4). The classification of individual isolates is shown in details in Supplementary Table S2.

**Table 4:** Partial results of partial least squares discriminant analysis, indicating for each source group the ten most important UNID-consensus spectra (ranked 1 to 10) that differentiated the isolates of the group from those of the other three groups.

Source group	Rank	No. of isolates					Replicate MS/MS spectra per isolate				Sequence tag
		Sewage	Cow	Dog	Pig	Total	Sewage	Cow	Dog	Pig	
Sewage	1	20	—	3	—	23	1.9	—	1.0	—	3
	2	18	—	4	4	26	4.3	—	1.0	1.5	7
	3	19	1	1	3	24	3.0	1.0	2.0	1.3	6
	4	19	—	4	4	27	3.3	—	1.0	1.5	4
	5	20	—	7	7	34	3.9	—	1.4	2.4	4
	6	16	—	2	3	21	1.9	—	2.0	1.3	5
	7	15	—	1	1	17	1.9	—	2.0	2.0	7
	8	19	—	10	7	36	3.6	—	1.4	2.0	5
	9	13	—	—	—	13	1.5	—	—	—	5
	10	17	—	9	6	32	4.5	—	1.6	1.8	5
Cow	1	1	19	—	2	22	1.0	4.5	—	2.0	5
	2	1	20	2	2	25	1.0	1.7	1.0	1.0	4
	3	1	18	—	1	20	1.0	1.8	—	1.0	6
	4	1	18	1	1	21	1.0	1.9	1.0	2.0	5
	5	2	19	—	5	26	1.5	2.1	—	1.2	11
	6	—	20	—	6	26	—	2.0	—	1.3	9
	7	3	20	2	8	33	1.0	1.7	1.0	1.0	6
	8	1	16	—	—	17	2.0	2.0	—	—	13
	9	—	17	—	3	20	—	1.9	—	1.0	6
	10	1	16	—	1	18	2.0	2.0	—	1.0	11
Dog	1	—	—	17	—	17	—	—	2.0	—	10
	2	—	—	17	—	17	—	—	5.9	—	5
	3	—	—	17	—	17	—	—	5.5	—	6
	4	—	—	17	—	17	—	—	1.7	—	7
	5	—	—	16	—	16	—	—	1.9	—	6
	6	—	—	16	—	16	—	—	19.8	—	5
	7	—	—	17	—	17	—	—	3.5	—	7
	8	—	—	16	—	16	—	—	1.8	—	5
	9	—	—	17	—	17	—	—	1.7	—	6
	10	—	—	16	—	16	—	—	2.3	—	11
Pig	1	1	5	6	13	25	1.0	1.2	2.0	3.4	7
	2	—	—	—	10	10	—	—	—	1.5	4
	3	—	3	4	13	20	—	1.7	2.5	3.1	7
	4	1	7	8	13	29	1.0	3.6	3.1	6.5	5
	5	—	1	2	12	15	—	3.0	1.5	2.3	4
	6	—	1	4	11	16	—	1.0	1.5	2.7	4
	7	—	2	4	12	18	—	5.5	4.3	6.1	4
	8	—	—	1	9	10	—	—	1.0	2.9	5
	9	—	—	1	10	11	—	—	2.0	1.7	3
	10	—	7	5	12	24	—	3.3	3.0	5.6	4

Data shown for each UNID-consensus spectrum are (i) the number of isolates that it was associated with in each source group, (ii) the average number of replicate MS/MS spectra derived from each associated isolate in a group, and (iii) the length of the longest sequence tag (i.e. no. of amino acids) identified using de novo sequencing.
