# Supplementary material for: A peptide identification-free, genome sequence-independent shotgun proteomics workflow for strain-level bacterial differentiation
Source: Sci Rep. 2015 Sep 23;5:14337. doi: 10.1038/srep14337 (PMC4585814; doi:10.1038/srep14337)
Supplement: Supplementary Information [file srep14337-s1.doc]

**Supplementary Information**

**A peptide identification-free, genome sequence-independent shotgun proteomics workflow for strain-level bacterial differentiation**

Wenguang Shao^a^, Min Zhang^b^, Henry Lam ^a,c^*, Stanley C. K. Lau ^b,d^*

Division of Biomedical Engineering^a^, Division of Environment^b^, Department of Chemical and Biomolecular Engineering^c^, and Division of Life Science^d^, The Hong Kong University of Science and Technology, Clear Water Bay, Hong Kong

*Address correspondence to: Stanley Lau (scklau@ust.hk) & Henry Lam (kehlam@ust.hk)

W.S. and M.Z. contributed equally to this work

**Supplementary Methods**

***E. coli* cultivation.** Each thawed stock of *E. coli* (10 μl) was inoculated to 1 ml of 3 % tryptic soy broth (TSB) and incubated overnight at 37 °C without shaking. Then 0.5 ml of the culture was transferred to 50 ml fresh TSB and grown to early stationary phase (O.D._600_ = 1.0) at 37 °C with shaking. Cells were collected for protein and DNA extraction after two times of washing in phosphate buffered saline with centrifugation (3000×g, 10 min).

**Peptide sample preparation.** Each cell pellet was lysed in a cell disrupter (25,000 psi) in 8 ml of phosphate buffered saline with 62.5 μM proteinase inhibitor (Roche Applied Science). Upon centrifugation (19,000×g, 30 min), an aliquot of supernatant with 20 μg protein (Abs_280nm_) was subjected to disulfide bond reduction using DL-dithiothreitol, the blocking of bond reformation using iodoacetamide, and subsequently trypsin digestion as previously described ^1^. After speed-vac drying (4 °C), the digest was dissolved in 0.1 % formic acid in water, desalted in C18 Resin Zip Tip (Merck Millipore). LC-MS grade water was from J. T. Baker. All other supplies and reagents were from Sigma-Aldrich, unless specified.

**LC-MS/MS analysis.** Solvents A and B in the mobile phase (flow rate: 150 μl min^-1^) were 0.1 % FA in water and 0.1 % FA in acetonitrile, respectively. Upon sample injection, the proportion of solvent B was raised linearly from 2 to 32 % in 30 min and further to 80 % in 2 min. After a 12 min isocratic step, solvent B was reduced to 2 % in 2 min. The injection of the next sample was made after a 9 min isocratic step. Five collision-induced dissociation MS/MS scans were taken for each full MS survey scan in data-dependent acquisition mode with dynamic exclusion (exclusion list size of 500, exclusion duration of 60 s, and repeat count of 2). The normalized collision energy was set to 35 %.

**DNA sample preparation.** Each cell pellet was suspended in 100 μL nuclease-free water and lysed by heating (94 °C, 20 min). Supernatants obtained after centrifugation (8000×g, 10 min) were measured spectrometerically. Samples passing the quality threshold of Abs_260nm_/Abs_280nm_ = 1.8 - 2.0 had nucleic acid concentrations adjusted to 50 ng μL^-1^ in nuclease free water before REP-PCR.

**Reproducibility of UNID- and ID-proteomic fingerprinting in source classification.** We repeated the analysis of isolate classification by substituting the UNID- and ID-proteomic fingerprint libraries with the MS/MS spectra generated from an additional set of culture of each dog isolate. The dog isolates were chosen for their RCC being neither 100 % nor the lowest among the four source groups in the UNID- and ID-proteomic fingerprint libraries (Table 3). We suppose that this would allow any upward or downward change in classification accuracy to be detected more easily in the reproducibility test.

An additional culture of each dog isolate was prepared using the methods and procedures above. This set of additional cultures served as a biological replicate of those of the dog isolates that were originally analyzed together with the isolates of the three other sources (Supplementary Fig. S1). As such, the additional cultures were made using a set of frozen stocks and a batch of culture medium that were different from those for the original set. The cell lysate of each additional culture was split into three equal portions as technical replicates prior to protein digestion (Supplementary Fig. S1).

The tryptic samples of the isolates in each set of technical replicates were injected to LC-MS/MS in a random order. The building of UNID- and ID-consensus spectra and their respective fingerprint libraries was repeated three times. In each iteration, the MS/MS spectra of the dog isolates in the original data set was substituted by those of one set of technical replicate. The effects of the data substitution on the performance of the UNID- and ID-proteomic fingerprint libraries in the differentiation of *E. coli* isolates of different sources were evaluated using Jackknife analysis.

**Supplementary Results and Discussion**

**Reproducibility of UNID- and ID-proteomic fingerprinting in source classification.** We prepared an additional set of cultures of the dog isolates (as biological replicates of the original set) and split them into three sets of technical replicates after cell lysis (Supplementary Fig. S1). Each set of technical replicates yielded 3.7 – 5.0 % fewer MS/MS spectra than the original set (Supplementary Table S3). However, the numbers of UNID- and ID-consensus spectra that were subsequently obtained for each set of technical replicates were generally higher than those of the original (Supplementary Table S3). Notably, the magnitude of difference (by percentage) between the technical replicates and the original was much larger for the ID-consensus spectra (21.2 – 25.8 %) than for the UNID counterpart (< 5.0 %) (Supplementary Table S3). These results indicate that, as far as the number of consensus spectra was concerned, similarity clustering of MS/MS spectra (UNID) yielded more consistent results than peptide identification (ID) for the dog isolates tested.

We created three replicate libraries of UNID- and ID-proteomic fingerprints by substituting, in three iterations, the consensus spectra of each set of technical replicates for those that were present in the original libraries (Supplementary Fig. S1). When the full set of consensus spectra were used (i.e. no filtering), all three replicate libraries of UNID-proteomic fingerprint attained the same ARCCs of 98.7 % whereas that of the original was 94.5 % (Supplementary Fig. S2). The difference was due to the three replicate libraries consistently having higher RCCs for the dog and pig source groups (Supplementary Table S4).

In comparison, the ARCCs of the ID counterparts (also full set of consensus spectra) varied in a wider range of 89.0 to 97.3 %, not only between technical replicates and the original but also the among the technical replicates themselves (Supplementary Fig. S2). Replicate library 1 (97.3 %) and 2 (95.9 %) had higher ARCCs than the original (91.8 %) as a result of the higher RCCs for the sewage and dog source groups (Supplementary Table S4). On the contrary, replicate library 3 had lower ARCC (89.0 %) than the original, primarily due to the higher frequency of misclassification (4 isolates) for the sewage source group (Supplementary Table S4).

Similar to the observation for the original library, the ARCCs of the replicate libraries of UNID- and ID-proteomic fingerprints changed over the sequential filtering of consensus spectra (Supplementary Fig. S2). The libraries of UNID-proteomic fingerprints varied relatively closely with each other; indeed, in many steps of filtering, their ARCCs were identical (98.6 %). On the contrary, much wider range of differences occurred among the original and the three replicates of the ID counterparts (Supplementary Fig. S2). In particular, the original and replicate 3 of the ID proteomic fingerprint libraries had their ARCCs increased after certain extent of consensus spectra filtering. But this was not the case for replicates 1 and 2 since their ARCCs were already relatively high without any filtering.

For both UNID- and ID-proteomic fingerprinting, the performances of the three technical replicates were generally closer to each other than they were to the original (Supplementary Fig. 2). The original and the three technical replicates were essentially biological replicates to each other (Supplementary Fig. 1); therefore, the differences observed between them is in part attributable to batch-to-batch variations in culture conditions that affect the types of protein expressed in different biological replicates of a bacterial isolate. The differences among technical replicates would be more likely associated with the inherent variability of the shotgun proteomics method ^2^. Such variability can be associated with the sample preparation steps (affecting the composition of peptides obtained from the cells) and/or the LC-MS/MS steps (e.g. chromatographic condition, ionization efficiency, instrument noise, and the stochasticity of the data-dependent acquisition scheme of MS/MS spectrometry) ^3^. Nonetheless, based on repeated testing of dog isolates, we observed that proteomic fingerprinting with consensus spectra constructed using similarity clustering (UNID) offered more consistent results in source classification than using peptide identification (ID). This may be attributed to the additional variability associated with the sequence searching step in the construction the ID-proteomic fingerprints.


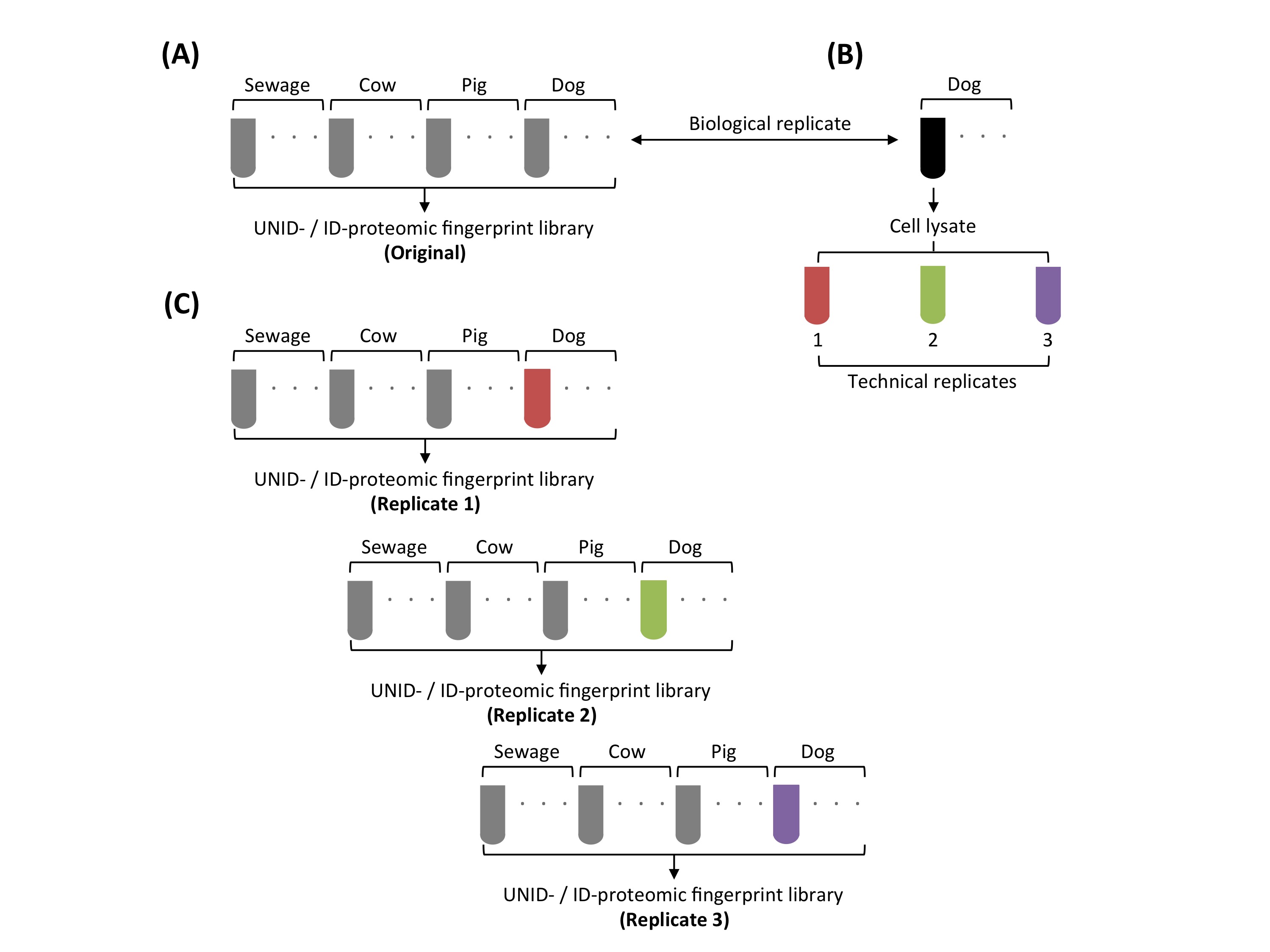


**Supplementary Figure S1** Experimental design to investigate the reproducibility of UNID-and ID-proteomic fingerprinting. The original UNID- and ID-proteomic fingerprint libraries were constructed using the MS/MS spectra obtained concomitantly from the *E. coli* isolates of all four fecal sources as described in the Material and Methods in the main text (A). To test for the reproducibility of the fingerprinting methods, an additional set of cultures of the dog isolates was prepared and served as the biological replicate of the cultures in the original libraries (B). The additional set of cultures was subjected to cell lysis. Each cell lysate was split into three technical replicates (B). The MS/MS spectra generated from each set of technical replicate was substituted for those of the dog’s in the original libraries in three iterations (C). A new set of UNID consensus spectra and a new fingerprint library were generated in each iteration (replicate 1, 2 and 3) c). The ARCCs of all libraries (the original and the three replicates) were determined using Jackknife analysis (Supplementary Fig. S2).


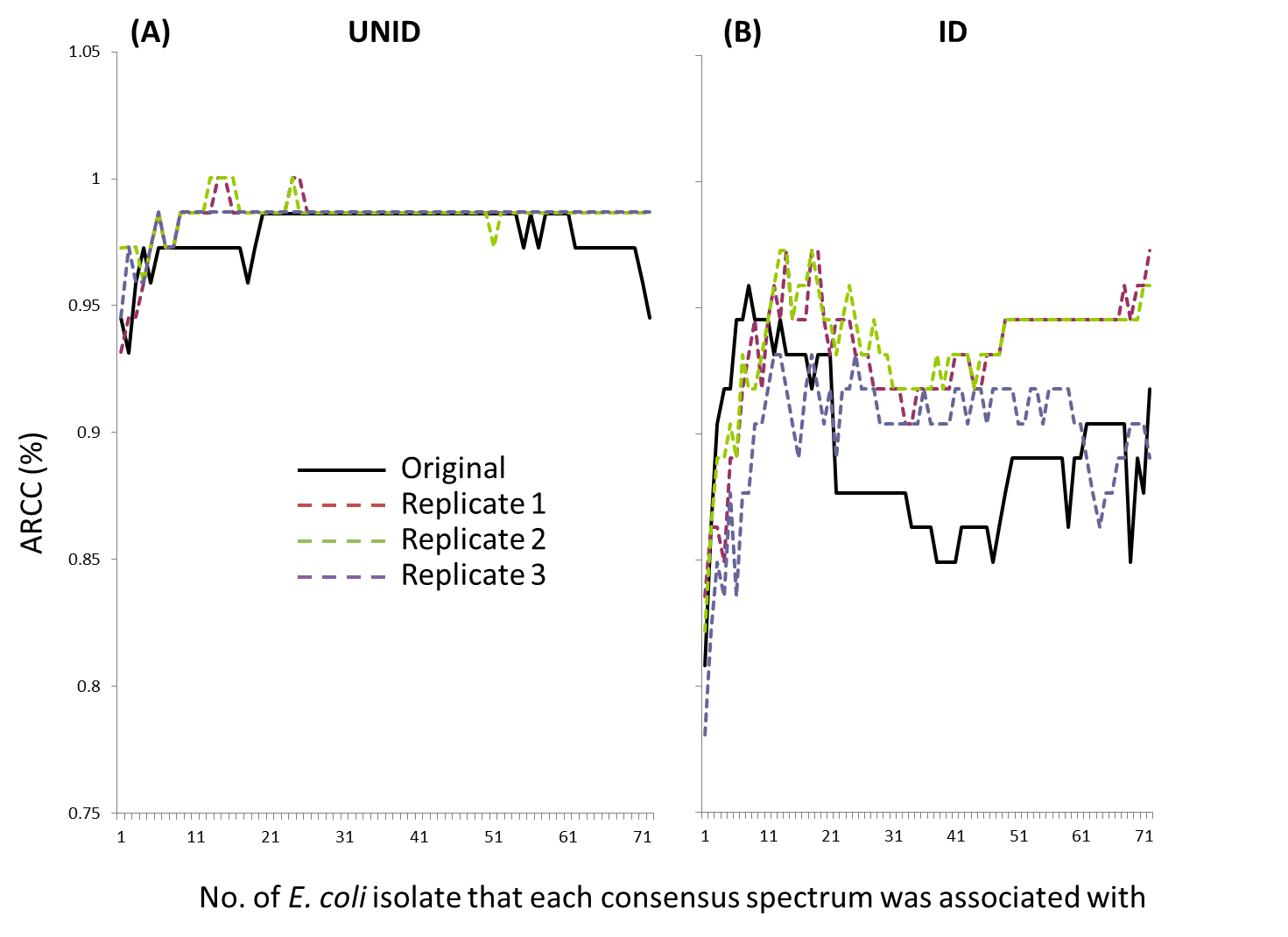


**Supplementary Figure S2** Reproducibility of UNID and ID proteomic fingerprinting in source classification of *E. coli* isolates. Data shown are the change in the average rate of correct classification (ARCC) by the original and the three replicates of (A) UNID- and (B) ID-proteomic fingerprint libraries as the consensus spectra were filtered according to the no. of *E. coli* isolates that they were associated with.

**Supplementary Table S1** Primers used in PCR verification of *E. coli*.

| Gene | Primer | Sequence (5’-3’) | Reference |
| --- | --- | --- | --- |
| lacY | EClpma (+1)  EClpma (-1) | GCACCTACGATGTTTTTGACCA  ACCAGACCCAGCACCAGATAAG | 4 |
| Cyd | cyd (+1)  cyd (-1) | CCGTATCATGGTGGCGTGTGG  GCCGGCTGAGTAGTCGTGGAAG | 4 |
| uidA | uidA (+1)  uidA (-1) | ATCGGCGAAATTCCATACCTG  GTTCTGCGACGCTCACACC | 4 |
| lacZ | lacZ (+1)  lacZ (-1 | ATGAAAGCTGGCTACAGGAAGGCC  GGTTTATGCAGCAACGAGACGTCA | 4 |
| 16S rRNA | ECA75F  ECR619R | GGAAGAAGCTTGCTTCTTTGCTGAC  AGCCCGGGGATTTCACATCTGACTTA | 5 |

**Supplementary Table S2** Source classification of *E. coli* isolates by REP-PCR, UNID-proteomic and ID-proteomic fingerprints. Data shown for the two proteomic fingerprinting methods are based on the complete set of consensus spectra (Fig. 4). Isolates that were misclassified are indicated by “v”. Isolates with no indication were correctly classified.

|  | Isolate | REP-PCR | | | |  | UNID-Proteomic | | | |  | ID-Proteomic | | | |
| --- | --- | --- | --- | --- | --- | --- | --- | --- | --- | --- | --- | --- | --- | --- | --- |
|  |  | Sewage | Cow | Dog | Pig |  | Sewage | Cow | Dog | Pig |  | Sewage | Cow | Dog | Pig |
| Sewage | 1 |  |  |  |  |  |  |  |  |  |  |  |  |  |  |
|  | 2 |  |  |  |  |  |  |  |  |  |  |  |  |  |  |
|  | 3 |  |  |  |  |  |  |  |  |  |  |  |  |  |  |
|  | 4 |  |  |  |  |  |  |  |  |  |  |  |  |  |  |
|  | 5 |  |  |  | v |  |  |  |  |  |  |  |  |  |  |
|  | 6 |  |  |  |  |  |  |  |  |  |  |  |  |  |  |
|  | 7 |  |  |  |  |  |  |  |  |  |  |  |  |  |  |
|  | 8 |  |  |  |  |  |  |  |  |  |  |  |  |  |  |
|  | 9 |  |  |  | v |  |  |  |  |  |  |  |  | v |  |
|  | 10 |  |  |  |  |  |  |  |  |  |  |  |  |  |  |
|  | 11 |  |  |  |  |  |  |  |  |  |  |  |  |  |  |
|  | 12 |  |  |  |  |  |  |  |  |  |  |  |  |  |  |
|  | 13 |  |  |  |  |  |  |  |  |  |  |  |  |  |  |
|  | 14 |  |  |  |  |  |  |  |  |  |  |  |  |  |  |
|  | 15 |  |  |  |  |  |  |  |  |  |  |  |  |  | v |
|  | 16 |  |  |  | v |  |  |  |  |  |  |  |  |  |  |
|  | 17 |  |  | v |  |  |  |  |  |  |  |  |  |  |  |
|  | 18 |  | v |  |  |  |  |  |  |  |  |  |  |  |  |
|  | 19 |  |  | v |  |  |  |  |  |  |  |  |  |  |  |
|  | 20 |  |  |  |  |  |  |  |  |  |  |  |  |  |  |
| Cow | 1 | v |  |  |  |  |  |  |  |  |  |  |  |  |  |
|  | 2 | v |  |  |  |  |  |  |  |  |  |  |  |  |  |
|  | 3 |  |  | v |  |  |  |  |  |  |  |  |  |  |  |
|  | 4 |  |  | v |  |  |  |  |  |  |  |  |  |  |  |
|  | 5 |  |  | v |  |  |  |  |  |  |  |  |  |  |  |
|  | 6 |  |  |  |  |  |  |  |  |  |  |  |  |  |  |
|  | 7 |  |  |  |  |  |  |  |  |  |  |  |  |  |  |
|  | 8 |  |  |  |  |  |  |  |  |  |  |  |  |  |  |
|  | 9 |  |  |  |  |  |  |  |  |  |  |  |  |  |  |
|  | 10 |  |  |  |  |  |  |  |  |  |  |  |  |  |  |
|  | 11 |  |  |  |  |  |  |  |  |  |  |  |  |  |  |
|  | 12 |  |  |  |  |  |  |  |  |  |  |  |  |  |  |
|  | 13 |  |  |  |  |  |  |  |  |  |  |  |  |  |  |
|  | 14 |  |  |  |  |  |  |  |  |  |  |  |  |  |  |
|  | 15 |  |  |  |  |  |  |  |  |  |  |  |  |  |  |
|  | 16 |  |  |  |  |  |  |  |  |  |  |  |  |  |  |
|  | 17 |  |  |  |  |  |  |  |  |  |  |  |  |  |  |
|  | 18 |  |  |  |  |  |  |  |  |  |  |  |  |  |  |
|  | 19 |  |  |  |  |  |  |  |  |  |  |  |  |  |  |
|  | 20 |  |  |  |  |  |  |  |  |  |  |  |  |  |  |
| Dog | 1 |  |  |  |  |  |  |  |  |  |  |  |  |  |  |
|  | 2 |  |  |  |  |  |  |  |  |  |  |  |  |  |  |
|  | 3 |  |  |  |  |  |  |  |  |  |  |  |  |  |  |
|  | 4 |  |  |  |  |  |  |  |  |  |  |  |  |  |  |
|  | 5 |  |  |  |  |  |  |  |  |  |  |  |  |  |  |
|  | 6 |  |  |  |  |  |  |  |  |  |  |  |  |  |  |
|  | 7 |  |  |  |  |  |  |  |  |  |  |  |  |  |  |
|  | 8 |  |  |  |  |  |  |  |  |  |  |  |  |  |  |
|  | 9 | v |  |  |  |  | v |  |  |  |  | v |  |  |  |
|  | 10 | v |  |  |  |  | v |  |  |  |  |  |  |  | v |
|  | 11 |  |  |  |  |  |  |  |  |  |  |  |  |  |  |
|  | 12 |  |  |  |  |  |  |  |  |  |  |  |  |  |  |
|  | 13 |  |  |  |  |  |  |  |  |  |  |  |  |  |  |
|  | 14 |  |  |  |  |  |  |  |  |  |  |  |  |  |  |
|  | 15 |  |  |  |  |  |  |  |  |  |  |  |  |  |  |
|  | 16 |  |  |  |  |  |  |  |  |  |  |  |  |  |  |
|  | 17 |  |  |  |  |  |  |  |  |  |  |  |  |  |  |
|  | 18 |  |  |  |  |  |  |  |  |  |  |  |  |  |  |
|  | 19 |  |  |  |  |  |  |  |  |  |  |  |  |  |  |
| Pig | 1 |  |  |  |  |  |  |  | v |  |  |  |  |  |  |
|  | 2 |  |  |  |  |  |  |  |  |  |  | v |  |  |  |
|  | 3 | v |  |  |  |  | v |  |  |  |  | v |  |  |  |
|  | 4 |  |  |  |  |  |  |  |  |  |  |  |  |  |  |
|  | 5 |  |  |  |  |  |  |  |  |  |  |  |  |  |  |
|  | 6 |  |  |  |  |  |  |  |  |  |  |  |  |  |  |
|  | 7 |  |  |  |  |  |  |  |  |  |  |  |  |  |  |
|  | 8 |  |  |  |  |  |  |  |  |  |  |  |  |  |  |
|  | 9 |  |  |  |  |  |  |  |  |  |  |  |  |  |  |
|  | 10 |  |  |  |  |  |  |  |  |  |  |  |  |  |  |
|  | 11 |  |  |  |  |  |  |  |  |  |  |  |  |  |  |
|  | 12 |  |  |  |  |  |  |  |  |  |  |  |  |  |  |
|  | 13 |  |  |  |  |  |  |  |  |  |  |  |  |  |  |
|  | 14 |  |  |  |  |  |  |  |  |  |  |  |  |  |  |

**Supplementary Table S3** The numbers of MS/MS spectra, UNID- consensus spectra and ID-consensus spectra obtained from the isolates of dog during the reproducibility test. Data shown were obtained from the set of dog isolates that were analyzed together with the isolates of the three other sources (original), and also from those that were prepared separately as three sets of technical replicates (replicate 1, 2 and 3). Numbers in parenthesis are percent increase (+) or decrease (-) in comparison to the original.

| Spectra | Original | Replicate | | |
| --- | --- | --- | --- | --- |
|  |  | 1 | 2 | 3 |
| MS/MS | 303605 | 292395 (-3.7) | 291661 (-3.9) | 288425 (-5.0) |
|  |  |  |  |  |
| UNID-consensus | 13585 | 13895 (+2.3) | 13687 (+0.8) | 13012 (-4.2) |
| ID-consensus | 3385 | 4259 (+25.8) | 4166 (+23.1) | 4103 (+21.2) |

**Supplementary Table S4** Reproducibility of UNID-proteomic and ID-proteomic fingerprinting in source classification of *E. coli* isolates. Data shown are the rate of correct classification by the original and the three replicate libraries (Supplementary Fig. S1) when the full sets of consensus spectra were used (Supplementary Fig. S2). The numbers indicate for each source group the percentage of its isolates being assigned to its own source (i.e. correct classification) and to each of the three other sources (i.e. incorrect classification) (“-“ = 0 %).

|  | UNID-Proteomic | | | | | | | | |
| --- | --- | --- | --- | --- | --- | --- | --- | --- | --- |
|  | Original | | | |  | Replicate 1/2/3 | | | |
| Source group | Sewage | Cow | Dog | Pig |  | Sewage | Cow | Dog | Pig |
| Sewage | 100 | - | - | - |  | 100/100/100 | -/-/- | -/-/- | -/-/- |
| Cow | - | 100 | - | - |  | -/-/- | 100/100/100 | -/-/- | -/-/- |
| Dog | 10.5 | - | 89.5 | - |  | -/-/- | -/-/- | 100/100/100 | -/-/- |
| Pig | 7.1 | - | 7.1 | 85.7 |  | 7.1/7.1/7.1 | -/-/- | -/-/- | 92.9/92.9/92.9 |
|  | ID-Proteomic | | | | | | | | |
|  | Original | | | |  | Replicate 1/2/3 | | | |
| Source group | Sewage | Cow | Dog | Pig |  | Sewage | Cow | Dog | Pig |
| Sewage | 90 | - | 5 | 5 |  | 100/95/80 | -/-/- | -/-/5.0 | -/5.0/15.0 |
| Cow | - | 100 | - | - |  | -/-/5.0 | 100/100/95.0 | -/-/- | -/-/- |
| Dog | 5.3 | - | 89.5 | 5.3 |  | -/-/5.3 | -/-/- | 100/100/94.7 | -/-/- |
| Pig | 14.3 | - | - | 85.7 |  | 14.3/14.3/14.3 | -/-/- | -/-/- | 85.7/85.7/85.7 |

**Refences**

1 Rodriguez, J., Gupta, N., Smith, R. D. & Pevzner, P. A. Does trypsin cut before proline? *J Proteome Res* **7**, 300-305 (2007).

2 Tabb, D.L., Vega-Montoto, L., Rudnick, P.A., et al. Repeatability and reproducibility in proteomics identifications by liquid chromatography-tandem mass spectrometry. *J Proteome Res* **9**, 761-776 (2010).

3 Piehowski, P.D., Petyuk, V.A., Orton, D.J., et al. Sources of technical variability in quantitative LC-MS proteomics: Human brain tissue sample analysis. *J Proteome Res* **12**, 2128-2137 (2013).

4 Horakova, K., Mlejnkova, H. & Mlejnek, P. Specific detection of *Escherichia* *coli* isolated from water samples using polymerase chain reaction targeting four genes: cytochrome bd complex, lactose permease, β‐d‐glucuronidase, and β‐d‐galactosidase. *J. Appl Microbiol* **105**, 970-976 (2008).

5 Sabat, G., Rose, P., Hickey, W. & Harkin, J. Selective and sensitive method for PCR amplification of *Escherichia coli* 16S rRNA genes in soil. *Appl Environ Microbiol* **66**, 844-849 (2000).
